# Health for the People: Past, Current, and Future Contributions of National Community Health Worker Programs to Achieving Global Health Goals

**DOI:** 10.9745/GHSP-D-20-00459

**Published:** 2021-03-31

**Authors:** Henry B. Perry, Stephen Hodgins

**Affiliations:** a Johns Hopkins Bloomberg School of Public Health, Baltimore, MD, USA.; b Editor-in-Chief, Global Health: Science and Practice Journal, and Associate Professor, School of Public Health, University of Alberta, Edmonton, Alberta, Canada.

## Abstract

National community health worker programs are at the dawn of a new era, given the growing recognition of their importance for achieving global health goals and for controlling the COVID-19 pandemic. Now is the time to provide them with the respect and funding that they need and deserve.

## Key Messages

- After almost a century of experience, innovation, adaptation, and evidence, national community health worker (CHW) programs are now recognized as one of the most valuable assets for reaching global health goals, including achieving universal health coverage and ending preventable child and maternal deaths by 2030.
- In 2019, the United Nations General Assembly called urgently to accelerate progress in achieving these global health goals recognizing that, at the current pace, these goals will not be achieved for up to one-third of the world's population.
- There is rapidly growing interest not only in CHWs but in community health more broadly, in engagement with communities for improving their own health, and in community-based surveillance for infectious disease outbreaks, especially now that the world is struggling to combat COVID-19 and is likely to face similar pandemics in the future.
- Training more professionalized CHWs with better and longer training, better supervision, improved logistical support, and well-defined career paths, and linking them to lower-level volunteer workers, each serving a small number of households, will help strengthen program effectiveness and improve CHW morale and long-term retention.

## INTRODUCTION

The year targeted for achieving the United Nations (UN) Sustainable Development Goals (SDGs)—2030—is now less than a decade away. The health-related SDG3 includes both universal health coverage (UHC) and ending preventable child and maternal deaths. But, at the current pace, the UN predicts that UHC will not be achieved for up to one-third of the world's population.^1^ In 2019, the UN General Assembly unanimously passed a resolution stating that “measurable acceleration is urgently needed” to reach the health-related targets of the SDGs by 2030.^1^ The United Nations also estimates that half the world's population (3.8 billion people) lacks access to essential health services.^1^ The UN High-Level Commission on Health Employment and Economic Growth estimates a shortfall of 18 million health workers to reach SDG targets.^2^

Without a major expansion of support for national community health worker (CHW) programs, such an acceleration is unlikely. The Director-General of the World Health Organization (WHO), Dr. Tedros Adhanom Gebreyesus, has affirmed “there will be no UHC without primary health care” (PHC).^3^ And we would add: in low-income settings, there can be no effective PHC without CHWs. Indeed, the coronavirus disease (COVID-19) epidemic has undermined PHC and health-related development work more generally, making more robust CHW programs that much more important to achieving health and development goals. This has been recognized by the new administration in the United States, with President Biden calling for hiring 100,000 new CHWs as part of his response to the epidemic.^4^

The COVID-19 epidemic has undermined PHC and health-related development work more generally, making more robust CHW programs that much more important to achieving health and development goals.

For many decades, governments and their partners—at national and global levels—have been trying to extend health care services and interventions to better reach the whole population, particularly those segments poorly served by existing health services. Clearly, health priorities and available resources vary across settings, but there have been common challenges:

- How to effectively (and efficiently) *make key lifesaving interventions available to the whole population* (e.g., immunizations, insecticide-treated mosquito nets, micronutrient supplementation, among many others)?
- How to overcome the challenges of *distance and geographic barriers* to extend services to segments of the population that cannot easily reach better equipped and staffed health centers and hospitals?
- How to *bridge cultural gaps* that may exist between educated health professionals—many originally hailing from urban settings—and those they are to serve?

In many settings, over the years, it has been evident that our current systems and approaches have not been fully able to meet these challenges and that there is a need for some kind of lower-level provider to bridge the gap at the community level.

## THE POTENTIAL CONTRIBUTION OF BROADER AND STRONGER NATIONAL CHW PROGRAMS FOR HELPING ACCELERATE PROGRESS TOWARD GLOBAL HEALTH GOALS

For achieving the health-related targets of the SDGs, there are 3 important reasons why CHWs are a better option, in many instances, than relying only on services provided at health facilities.

1. CHWs can be trained and deployed quickly. Although building more PHC facilities and training and deploying physicians, nurses, and other health care professionals is certainly needed, in many cases this by itself will not accelerate progress, in the near term, simply because of the lag time required for training and deploying higher-level workers and building the PHC facilities where they can work.
2. Since CHWs are based in the community, the challenges of geographical access are greatly reduced. Many lower-income countries with dispersed populations have insufficient transportation infrastructure to enable all their citizens to readily access health facilities; people in many rural settings still travel mostly by foot. In such countries, achieving equitable access for all to PHC centers will require the construction and staffing of an extraordinary number of additional facilities. Given the markedly lower use of health facilities among those living more than 45 minutes from a health facility,^5^ in most low- and middle-income countries it will not be feasible, over the short- to medium- term, to achieve 90% population coverage using only PHC center and hospital platforms. Furthermore, even with a marked increase in the number of such facilities, it will remain challenging to recruit and retain the needed medical and nursing staff needed in more isolated areas in low-income countries. In contrast, CHW programs use locally available human resources; supporting and improving availability of appropriately-trained CHWs would cost less and result in quicker and more significant improvements in access to basic care and to preventive and promotive services.
3. Given current disease burden, more lives of mothers and children can be saved by comparatively simple, community-based interventions than those requiring a PHC center or a hospital. According to recent modeling,^6^ expanding and increasing coverage of services that can be provided by CHWs in the community could avert 2.3 million maternal and child deaths per year. Contrast this with the 0.8 million averted deaths achieved by increasing coverage of services requiring higher-level workers in a PHC facility and 0.9 million by scaling up services requiring hospital-level care.^6^ These estimates do not take into account the additional impact that could be achieved by having CHWs providing family planning services in the community and at household level. Given the well-documented capability of well-supported and supervised CHWs to competently provide family planning services, extending services in this way reduces the unmet need for contraception, with many benefits not only for health but also for other areas of development.^7^

Aside from the well-established role of CHW programs in maternal and child health, it is becoming increasingly evident that CHWs can play an important role in screening and managing noncommunicable diseases, such as hypertension and diabetes,^8^ and identifying and following up patients needing basic and essential surgical care, increasingly recognized as a major global health priority.^9^ Task shifting and extending care beyond facilities are critical for increasing health coverage. Furthermore, including CHWs as integral members of PHC teams can potentially strengthen the PHC system and improve quality of care by facilitating access to higher levels of care and following up patients after treatment at a higher-level facility.

Including CHWs as integral members of PHC teams can potentially strengthen the PHC system and improve quality of care by promoting more effective use of higher levels of care.

The Joint United Nations Program on HIV/AIDS (UNAIDS) has called for urgent new investments to be made in recruiting, training, and deploying 2 million additional CHWs to ensure Africa's success in ending AIDS as a public health threat and in attaining sustainable health for all of Africa.^10^

In a 2018 report,^11^ the Lancet Global Health Commission on High Quality Health Systems points out that deficiencies in the quality of care provided to patients currently using the health care system are responsible for 5.2 million deaths each year that could be prevented by high-quality health care. Appropriately, the Commission concludes that improving the quality of care in existing health systems is an urgent priority for resource-constrained settings. The Commission did not, however, address the question of how health systems can reduce the 3.2 million deaths that it documents are due to nonutilization of the health system. It is important to recognize that adverse outcomes associated with nonuse are disproportionately concentrated among the poor.^12^ Improving the quality of care provided by the existing hospital-centric health system is unlikely, on its own, to be sufficient to ensure universal access or achieve optimal population health outcomes. In most resource-constrained settings, today, facilities are too far away for a significant portion of the population. In low-income settings, recourse to health facility services diminishes exponentially with distance from the facility and declines precipitously at 3 kilometers, or more than a 45-minute walk from the facility.^5^^,^^13^ So, equity considerations call for attention to access; CHWs represent an effective and cost-efficient strategy for addressing this challenge.

Numerous studies have documented that those making use of health facility services are better off financially and educationally than the population as a whole^14^; prioritizing public investment at health facility-level, particularly if focused on hospitals, disproportionately benefits the better-off. Persisting disparities, by household level of wealth, in coverage of essential maternal-child interventions (as well as other basic health services, including family planning, that CHWs can provide) call for attention not only to the quality of services but also to access, for the poorest segments of society.

With appropriate support, CHWs are able to provide competent lifesaving prevention and treatment services for many conditions (including malaria, postpartum hemorrhage, childhood pneumonia, diarrhea, and acute malnutrition, among many others).^15^ They can also direct patients and their families to appropriate sources of care and accompany them to the health facility^16^ or facilitate engagement with health services in other ways. CHWs can thus help create greater community trust in the health system.

As Gwatkin et al.^17^ observed more than 40 years ago:

*Unless services reaches those most in need, even the best-conceived programs can … have little impact on mortality. Thus, … the development of plans for getting services to the people is as important as are decisions concerning which services should be offered.*

One might add … getting basic and essential services to people as important as the quality of those services. In summary, although there is now a well-recognized need for improving the quality of existing services in health systems, improving health outcomes at the scale of whole populations will also require improved access, which can be achieved through robust CHW programs.

## THE NEED TO INVEST MORE IN NATIONAL CHW PROGRAMS

The UN has determined that UHC will be required to achieve all the SDGs by 2030, not just health-related SDG3, and that to achieve UHC, governments will need to invest an additional 1% of their GDP in expanding and strengthening their PHC systems.^18^ The UHC conceptual framework focuses on financial “coverage” of the costs of clinical services. However, the concept of UHC also needs to be understood to include the provision of essential preventive, promotive, and curative services to everyone requiring them, particularly those with the greatest needs. To expand access to essential services a significant proportion of the investment at the PHC level needs to be directed to strengthening national CHW programs, generally with free provision of services. These services need to specifically target the poor and socially vulnerable and those living in more remote locations. CHWs represent a key strategy to reduce health inequities.

To expand access to essential services, a significant proportion of the investment at the PHC level needs to be directed to strengthening national CHW programs, generally with free provision of services.

However, despite robust evidence of the effectiveness of CHWs in delivering high-impact interventions,^15^ country investments in such programs, to date, are only a small fraction of what has been spent on PHC more broadly. Furthermore, only 2.5% of official development assistance for health over the past decade has specifically targeted CHW programs and fully two-thirds of this has been for vertical programs related to HIV/AIDS, malaria, TB, reproductive health, or family planning.^19^ A recent report from the Center for Accelerating Innovation and Impact and the Financing Alliance for Health estimated that an additional US$2 billion is needed annually to build and strengthen CHW programs in sub-Saharan Africa, alone.^20^

Finally, national CHW programs are beginning to garner the attention and recognition they deserve, as an integral component of PHC.^21^ In 2018, the WHO adopted the *WHO Guideline on Health Policy and System Support to Optimize Community Health Worker Programs*.^22^^,^^23^ The following year, the World Health Assembly passed an historic resolution on CHWs, highlighting their role in ensuring “that UHC and comprehensive health services reach difficult-to-access areas and vulnerable populations,” and in “advancing equitable access to safe, comprehensive health services.” The Assembly called on member states to “optimize” community health worker programs as part of the global strategy to achieve UHC and SDG3.^24^

## THE NEED FOR MORE INFORMATION ABOUT NATIONAL CHW PROGRAMS

Despite their importance, information currently available on national CHW programs is sparse and not easily accessed. In 2010, the Global Health Workforce Alliance and the WHO released a comprehensive report on the global experience with national CHW programs, which included a systematic review of literature on this topic and a set of 8 case studies, 3 from Africa, 3 from Asia, and 2 from Latin America.^25^ In 2014, a group of experts produced a guide on developing and strengthening CHW programs at scale, which drew heavily on lessons from large-scale CHW programs.^26^ In 2017, this group produced a set of 13 case studies of national CHW programs.^27^ The program Advancing Partners and Communities subsequently produced a series of case studies of 25 national CHW programs, with a particular focus on family planning and HIV/AIDS services.^28^ More recently, informative case studies have been developed on several national CHW programs.^29^

Over the years, many countries have made use of community-level providers to extend the reach of their health services. Through the 1960s and 1970s, the largest-scale program efforts in global health focused on specific infectious diseases—malaria, smallpox, and TB—and it was common to employ locally recruited outreach workers who were quickly trained and deployed for program-specific, community-level work; there were few large-scale examples of integrated CHW programs (China's barefoot doctor program being a notable case).^30^ In various regions of the world there were, however, small-scale, NGO-run programs (e.g., Jamkhed^31^^,^^32^ and SEARCH/Gadchiroli^33^^–^^35^, both in India) that were having notable health impact and exemplified key principles that could inform larger-scale efforts. A vital insight was that community-level workers needed to be: (1) accepted and respected by the community; and (2) well-connected to and supported by the PHC system, including reliably supplied with needed commodities.

The 1978 Declaration of Alma-Ata marked a global-level recognition of the potential for CHWs to serve as a foundation for PHC. Inspired by this vision, beginning in the early 1980s, many countries established national CHW programs. However, over the years, important lessons from earlier national CHW program experiences were often ignored. Instead, many saw CHWs as a panacea, a way of delivering PHC on the cheap. As a result, many programs were inadequately supported and experienced high attrition.^36^ By the late 1980s, the pendulum had swung away from CHW programs. Two seminal books—*The Community Health Worker: Effective Programs for Developing Countries*^37^ and *Community Health Workers in National Programs*^38^—cast a sharp critical eye, drawing lessons from large-scale CHW programs implemented over the preceding decade. Both books drew attention to systems support needed for CHW programs to be effective as a part of robust PHC services operating at scale.

Over the years, important lessons from earlier national CHW program experiences had often been ignored.

In the following decades, there have been further cycles of waxing and waning of enthusiasm for CHW programs. Today, it is recognized that CHWs have potential to play an important role in PHC and to contribute to more rapid progress in population health status improvements. Certainly, as we have seen in the past, shifting tasks or functions typically assumed by physicians or other highly trained professionals to auxiliary health workers and CHWs can make such services available to segments of the population that otherwise have considerable difficulty accessing care. CHW and health auxiliary programs have played and will continue to play an important role in delivering key interventions to rural populations, including: immunization, antenatal and postnatal care, family planning, management of childhood illness, malaria prevention and treatment, and nutrition-related services. However, with urbanization, rising levels of education, technological innovations (notably the now nearly universal use of cell phones), and an epidemiologic shift toward a proportionally greater burden of noncommunicable diseases, the needs of the population and optimal strategies to address those needs will continue to evolve.

CHWs should not be seen as a stopgap; they can and should be a key strategy for strengthening PHC. Although we may think of these programs as a strategy for low-income countries, there are many examples of CHWs playing helpful roles in high-income countries, as brokers between the community and health services.^39^^,^^40^ CHWs can be part of strategies for the *future* of PHC. The need for community-level workers for the response to the current COVID-19 pandemic is a case in point. As we noted earlier, this has been recognized at the highest levels, with U.S. President Joseph Biden having recently called for the hiring of 100,000 CHWs to support the COVID-19 response in the US.^4^

CHWs should not be seen as a stopgap; they can be part of strategies for the future of PHC.

In global health, we see ourselves as making evidence-based decisions, but an important type of evidence is often neglected: evidence arising from past (and current) *program experience*. As a consequence of this blind spot, we fail to apply insights garnered from analogous experiences in other settings, and we unnecessarily repeat mistakes or struggle, reinventing the wheel.

## RECENTLY RELEASED COMPENDIUM OF NATIONAL CHW CASE STUDIES

A recently-released resource, *Health for the People: National Community Health Worker Program from Afghanistan to Zimbabwe*, ^41^ provides insights into national CHW programs—their structure, their achievements, and the challenges they face. This book comprises 29 case studies of national CHW programs from: Afghanistan, Bangladesh (BRAC's world-renowned CHW program being the 1 nongovernmental organization program included), Brazil, Ethiopia, Ghana, Guatemala, India, Indonesia, Iran, Kenya, Liberia, Madagascar, Malawi, Mozambique, Myanmar, Nepal, Niger, Nigeria, Pakistan, Rwanda, Sierra Leone, South Africa, Tanzania, Thailand, Uganda, Zambia, and Zimbabwe. Each case study has at least 1 author who has personal in-country experience with the program described and follows a common format, helping facilitate comparisons across cases. The case studies look at a mix of CHW types, from the more professionalized end of the spectrum to less formalized community health volunteer programs. The programs described are drawn from diverse regions and both low- and middle-income countries. The publication is timely, given the growing interest not only in CHWs but also in community health services more broadly, in engaging communities for improving their own health, and in community-based surveillance and other forms of support for priority infectious disease outbreaks (especially now as we struggle to combat COVID-19).

### Insights From Country Program Experiences

As is evident on review of these program case studies, the circumstances across these 29 countries vary considerably regarding:

- Geographic and demographic factors
- Responsibilities assumed by CHWs
- Robustness of government health services and their support systems
- Role of private providers

There is no single, one-size-fits-all model for CHW programs. Some employ full-time paid CHWs, others offer allowances and other incentives and expect only part-time service, and yet others engage volunteers having only intermittent functions (e.g., as mobilizers or distributors in twice-annual child health days). In almost all the programs reviewed, part of the role the CHW serves is as an intermediary between the community and the government PHC system, encouraging and supporting use of these services as well as adoption of healthy practices (e.g., exclusive breastfeeding and use of insecticide-treated nets). In some programs, CHWs play a role dispensing or resupplying health-related commodities (e.g., oral contraceptive pills, sachets of oral rehydration salts, or micronutrient supplements). In other programs, CHWs have assumed responsibility for functions—under task shifting^42^—that are normally performed by more highly trained health workers, including assessing for and treating childhood illness and administering injectable contraceptives. Especially for communities lacking easy access to facility-based services, provision of such services through CHW outreach can help close an important coverage gap. Also, offering comparatively simple services at the community level (for example, resupplying oral contraceptive pills or antenatal iron supplements) can relieve some of the volume of clients who could otherwise overburden health centers.

Program experience has shown, nevertheless, that even simple community-based services provided by volunteers or very modestly compensated CHWs can only be reliably delivered if there are functional support systems, and these require a significant financial commitment.

Program experience has shown that even simple community-based services provided by CHWs can only be reliably delivered if they are adequately supported, requiring functional systems and significant financial commitment.

Review of the programs documented in this set of case studies reveals that all of these programs exist in dynamic, evolving health systems, many of which are quite pluralistic. As education levels rise, in many countries there are increasing numbers of credentialed health workers available that can be deployed to peripheral-level health services where, previously, only minimally trained CHWs were serving. Necessarily, this has resulted in evolving roles of the various categories of health workers and CHWs present. Epidemiologic and demographic transitions have also resulted in new roles for such workers, including important, previously neglected conditions such as noncommunicable diseases and mental health. As we have noted, private providers are now an important source of health services in many countries where CHW programs are active. In some countries, there are private sector providers, recognized as “village doctors,” “rural medical practitioners,” “patent medicine vendors,” or “medicine shop providers,” who may have the same training and credentials as government CHWs or auxiliary health workers, and may—in fact—be engaged in dual practice, working both in the private sector and in government PHC services. Effective strategies at the community level need to take into account the real situation on the ground rather than be based on an assumed, hypothetical situation. In some countries, ministries of health and development partners have actively engaged with private and informal sector providers, often complementing efforts undertaken through CHW programs. Under social-marketing and social-franchising schemes, for example, the presence of such providers has been leveraged for a range of programs.^43^^,^^44^

## TOWARD A FUTURE AGENDA

There are many reasons to be optimistic that large-scale CHW programs are at the dawn of a new era. Drawing lessons from programs documented in the recently released compendium of national CHW case studies, and others, we and our broader team have developed a soon-to-be published journal supplement that identifies important challenges to be overcome for these programs to reach their full potential. The summary article^45^ calls for:

1. Broader recognition of the value of CHW programs as the foundation of effective health system function and greater respect for CHWs as indispensable members of the PHC team
2. Funding that is steady, growing, sustainable and progressively less donor-dependent
3. Better remuneration for CHWs
4. Stronger supervision and logistical support
5. Rigorous ongoing monitoring and evaluation and independent academic research to support continuous improvement
6. Allocation of broader CHW roles and tasks that CHWs can competently perform (as confirmed by rigorous evaluation), including for noncommunicable diseases as well as for surveillance, detection of disease outbreaks, vital events registration, and care navigation (by accompanying patients for services at facilities) and expansion of the number of CHWs to prevent work overload and ensure population coverage
7. Greater flexibility in programming to respond to local health needs that are identified by working in partnership with communities

Achieving these conditions for optimal program effectiveness will require the cultivation of strong leadership within countries as well as greater recognition within the global health community that the expansion and strengthening of CHW programs are critical for achieving the SDGs and other global health goals, including “Health for All.”

Given that CHWs have long been seen as a promising resource for strengthening health care in resource-constrained settings, on what basis do we propose that we are on the threshold of a new era for CHW programs? We argue that several historical trends are now converging that will enable CHW programs to cross the threshold.

There is extensive research evidence^15^ and national experience from exemplar countries, such as Bangladesh, Brazil, Ethiopia,^29^ and Nepal,^46^ demonstrating that CHWs can and, in fact, do make important contributions to population health improvement. Furthermore, given persisting inequities in health outcomes and service utilization between and within countries, fundamental health systems changes are needed for current and future global health goals to be achieved. CHW programs should not be dismissed as merely a temporary solution for a problem in low-income settings that will go away (i.e., to reduce the burden of disease among mothers and children and from communicable disease). Instead, such programs must be recognized as an important permanent element needed for any health system anywhere to achieve its full potential. Indeed, this acknowledgement is reflected in the growing use of CHWs in middle-income countries such as Brazil^47^ and high-income countries such as the United States.^48^ Finally, the COVID-19 pandemic has made it abundantly clear—as did the 2013–2016 Ebola outbreak in West Africa^49^—that CHWs have a critical role to play in surveillance, case detection and effective frontline response.^50^ They will have a critical role to play in COVID-19 vaccination efforts. National CHW programs are being proposed for Great Britain and the United States in part to respond to the current crisis but also to provide a permanent new cadre of health workers to address other unmet health needs.^4^^,^^51^

## CONCLUSION

Small, innovative, proof-of-concept programs can certainly generate rich and important lessons; indeed, such programs served as inspiration for the 1978 Declaration of Alma-Ata. However, programs at national scale face a different set of challenges. For that reason, case studies of such programs—providing useful information on CHW roles and performance, as well as on context and systems support—can be particularly relevant and useful for national-level policy makers and program managers concerned about delivery of services to whole populations.

Although there has been increased attention to CHW programs over the past several years, challenges remain in ensuring adequate systems support for CHWs. Despite nearly a century of experience, we continue to struggle with how to define, deploy, and support such health workers. Almost 30 years ago, in his introduction to a series of case studies of national CHW programs, Stephen Frankel wrote^36^:

*There is no longer any place for discussion of whether CHWs can be key actors in achieving adequate health care. The question is how to achieve their potential*.

His statement is equally true today.

CHWs count. It is time, now, for governments and UN agencies to: enumerate CHWs—together with doctors, nurses, and allied health personnel—in their official health statistics; ensure they are reflected in national planning for human resources for health, including provision of adequate numbers of CHWs; and give priority to supervisory, logistical, and other needed support for these programs.

Despite the obstacles these programs have often faced, the future for CHW programs appears to be bright. Finding new sources for financing will be important for building stronger programs. Training more professionalized CHWs, providing better supervision, strengthening logistical support, offering well-defined career paths, and (in some settings) linking them to lower-level volunteer workers, each serving a small number of households, will result in more effective programs and improved CHW morale and retention.

The time for national CHW programs as an underfunded afterthought has passed. Millions of lives are at stake.

## Notes

**Peer Reviewed**

**First published online:** February 10, 2021.
